# Supplementary material for: MetaRibo-Seq measures translation in microbiomes
Source: Nat Commun. 2020 Jun 29;11:3268. doi: 10.1038/s41467-020-17081-z (PMC7324362; doi:10.1038/s41467-020-17081-z)
Supplement: Supplementary file 10 — Supplementary Data 7 [file 41467_2020_17081_MOESM10_ESM.zip › File2/Confidence_VeryHigh_Taxonomy/107693_out.krona.html]

Javascript must be enabled to view this page.

members
magnitude
magnitudeUnassigned
count
unassigned
taxon
rank

107693\_out

24

24
2
superkingdom

24
phylum
1239

24
class
186801

24
186802
order

24
family
541000

genus
1263
24

4

SRS011084\_contig\_number\_contig-100\_1258.197295SRS014235\_contig\_number\_49350SRS019685\_contig\_number\_28544SRS893230\_contig\_number\_contig-100\_1403.77813
species
2293178

5

SRS016018\_contig\_number\_13194SRS024492\_contig\_number\_contig-100\_1957.106646SRS042628\_contig\_number\_3063SRS045826\_contig\_number\_13430SRS143523\_contig\_number\_3006
species
2293216


SRS015663\_contig\_number\_contig-100\_2192.202757SRS051031\_contig\_number\_contig-100\_2657.185479
2
2293161
species

2293149
species
13

SRS013965\_contig\_number\_41270SRS017521\_contig\_number\_contig-100\_9774.186143SRS018313\_contig\_number\_11225SRS019808\_contig\_number\_contig-100\_555.117063SRS020869\_contig\_number\_38408SRS048870\_contig\_number\_21929SRS054905\_contig\_number\_contig-100\_796.154534SRS074964\_contig\_number\_2417SRS1041090\_contig\_number\_3276SRS104912\_contig\_number\_19416SRS140492\_contig\_number\_contig-100\_656.206830SRS142923\_contig\_number\_24097SRS148424\_contig\_number\_contig-100\_828.196695
